# Supplementary material for: A smartphone microscopic method for rapid screening of cloth facemask fabrics during pandemics
Source: PeerJ. 2020 Jul 30;8:e9647. doi: 10.7717/peerj.9647 (PMC7396138; doi:10.7717/peerj.9647)
Supplement: Supplemental Information 2 — Figure S1 and S2 show type I and type II facemask fabrics, respectively. [file peerj-08-9647-s002.pdf]

Supporting information for

A smartphone microscope method for rapid screening of cloth facemask fabrics during pandemics

Bhanu Bhakta Neupane[1,2*], Ravindra Kumar Chaudhary[3], Amita Sharma[2]

1) Central Department of Chemistry, Tribhuvan University, Kathmandu Nepal
2) Center for Analytical Sciences, Kathmandu Institute of Applied Sciences, Kathmandu, Nepal
3) Department of Chemistry, Amrit Campus, Tribhuvan University, Kathmandu Nepal

***Corresponding author**
Bhanu Bhakta Neupane
Central Department of Chemistry, Tribhuvan University, Kathmandu Nepal
Emial address: bbneupane@cdctu.edu.np

Supporting figures

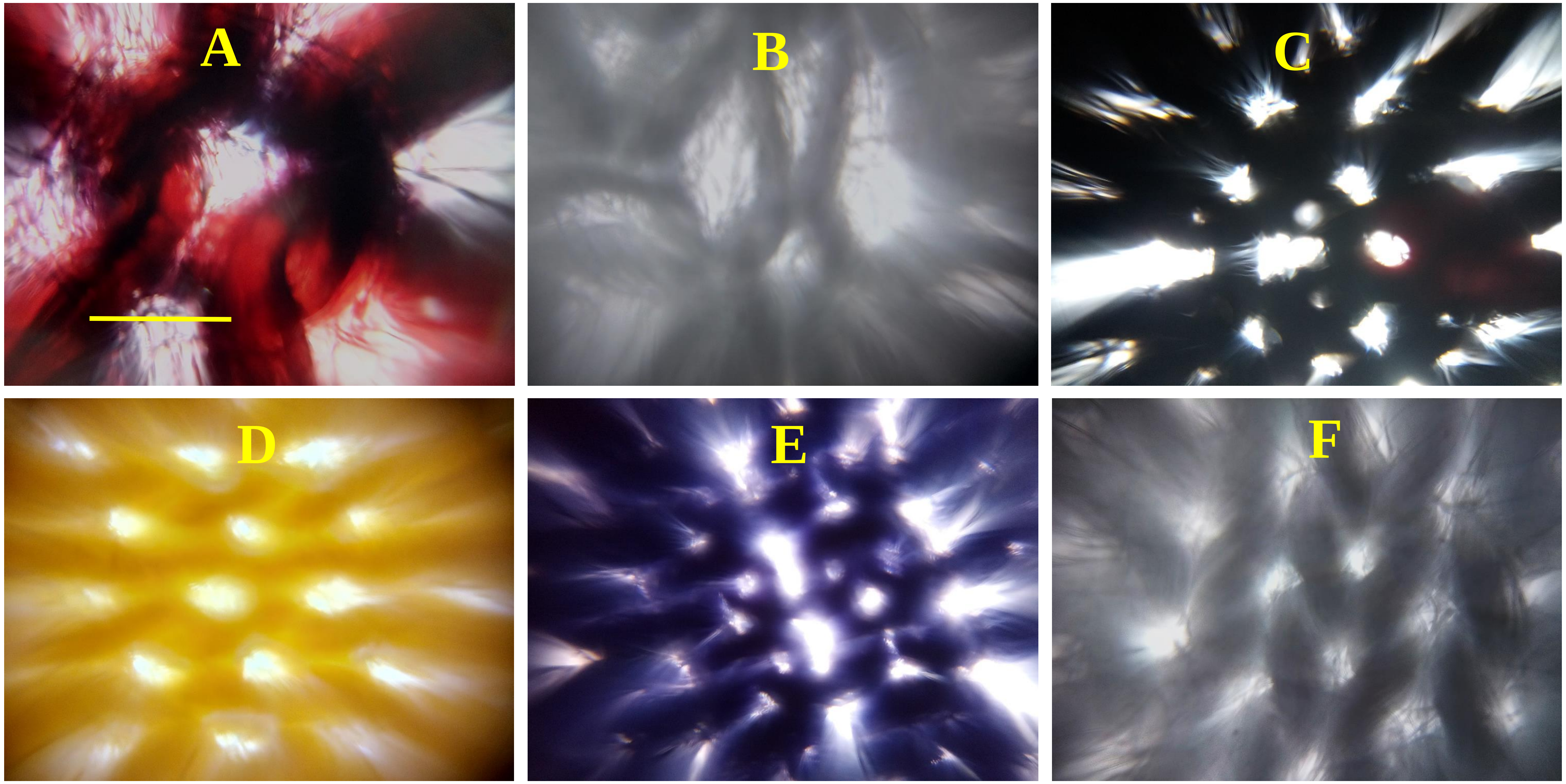

Supporting figure 1: Representative images of type I cloth facemask. (A) M3, (B) M4, (C) M7, (D) M8, (E) M9, and (F) M11. The scale bar shown in A is 400 micrometer and applies to all images.

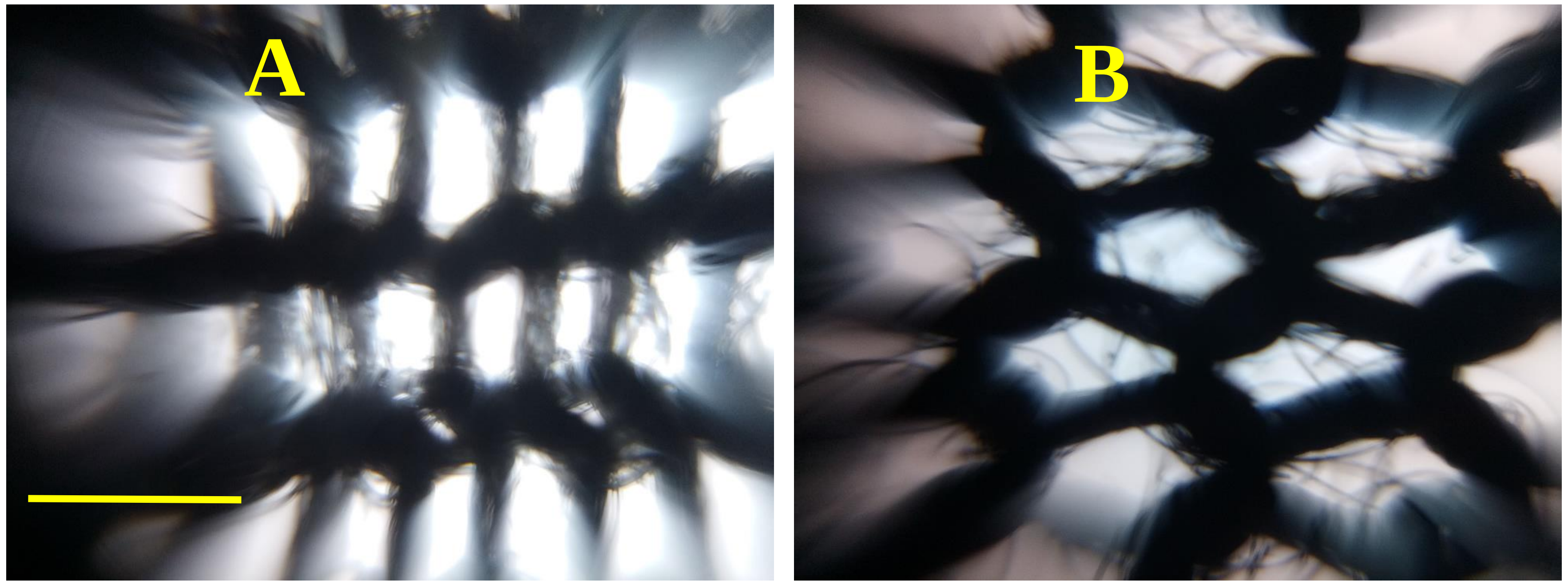

Supporting figure 2: Representative images of type II cloth facemask. (A) layer I (B) layer II of M14. The scale bar shown in A is 400 micrometer and applies to image B also.
